# Decoding electroacupuncture’s efficacy: a comparative study on gut microbiota and metabolic profiles in C57 and 3xTg-AD mice

**DOI:** 10.3389/fmicb.2025.1687697

**Published:** 2026-01-16

**Authors:** Chuyu Deng, Xingyu Cao, Xueying Wang, Chunzhi Tang, Zhenhua He

**Affiliations:** 1 Department of Pediatric Neurosurgery, Guangdong Women and Children Hospital, Guangzhou, China; 2 Women and Children’s Hospital, Southern University of Science and Technology, Shenzhen, China; 3 Clinical Medical College of Acupuncture, Moxibustion and Rehabilitation, Guangzhou University of Chinese Medicine, Guangzhou, China

**Keywords:** 3xTG-AD mice, AD, electroacupuncture, gut microbiota, T2DM

## Abstract

**Objective:**

This study aimed to investigate the impact of electroacupuncture (EA) on blood glucose, lipid profiles, and gut microbiota in WT (C57BL/6J) and 3xTg-AD mice with type 2 diabetes mellitus (T2DM) by examining fecal microbiota.

**Methods:**

C57BL/6J and 3xTg-AD mice were randomly divided into six groups (*n* = 12 per group) using a random number table: normal diet (C57-ND, 3X-ND), high-fat diet (C57-HFD, 3X-HFD), and high-fat diet with electroacupuncture (C57-EA, 3X-EA). Mice were fed either a high-fat or standard diet for 16 weeks, followed by electroacupuncture treatment at the ST36 acupoint. Following the intervention, body weight, fasting blood glucose (FBG), glucose tolerance test (GTT), and serum lipid levels were tracked. The analysis of fecal microbial communities was conducted through 16S rDNA sequencing.

**Results:**

EA treatment significantly reduced body weight gain in C57 T2DM mice, but not in 3xTg-AD T2DM mice. It decreased the area under the curve (AUC) of the glucose tolerance test (GTT) after 16 weeks and improved lipid profiles by reducing total cholesterol (TC), triglycerides (TG), and low-density lipoprotein cholesterol (LDL-C), while increasing high-density lipoprotein cholesterol (HDL-C) in both mouse models. Overall, EA demonstrated a more pronounced therapeutic effect in C57 mice compared to 3xTg-AD mice. In both types of mice, the EA group had a higher ASV abundance compared to the HFD group. EA improved α-diversity and β-diversity, and the therapeutic efficacy were impaired in 3xTg-AD mice. Microbial diversity, richness, and composition varied between 3xTg-AD and C57 mice. In 3xTg-AD mice, there was an increase in the relative abundance of *Deferribacterota* (phylum) and *Campylobacter* (class), while *Proteobacteria* (phylum), *Bacilli* (class), *Oscillospira*, and *Rikenellaceae* (family) showed a decrease. The composition changes induced by a high-fat diet (HFD) differed between 3xTg-AD and C57 mice. Electroacupuncture (EA) treatment significantly increased the *Ruminococcaceae* family in C57 mice and the *Monoglobaceae* family in 3xTg-AD mice.

**Conclusion:**

EA significantly enhanced glucose metabolism and lipidemia in HFD C57 mice, but its therapeutic impact was reduced in 3xTg-AD mice. The variation in gut microbiota regulation in 3xTg-AD mice could account for the response to EA treatment.

## 1 Introduction

Obesity has turned into a rising global public health concern over the last few decades. Globally, nearly 2 billion adults are overweight, with over half classified as obese (Hoffman et al., 2021). Additionally, there is a rising body of evidence that suggests diet and nutrition can be altered to influence the development and advancement of Alzheimer’s disease (AD) (Stefaniak et al., 2022). Obesity is linked to metabolic conditions such as hyperlipidemia, hypercholesterolemia, cardiovascular disease, liver steatosis, and type 2 diabetes mellitus (T2DM), all of which increase mortality risk (Cheong et al., 2025). A high-fat diet (HFD) is associated with metabolic issues like obesity, systemic inflammation, dyslipidemia, insulin resistance (IR), and increased glucose levels, potentially worsening neurodegenerative processes related to AD (Czech, 2017). Hence, the effective management of obesity and T2DM is essential in preventing and controlling chronic non-communicable diseases.

Studies using 3xTg-AD mouse models indicate that consuming HFD intensifies cognitive deficits (Gannon et al., 2022; Natale et al., 2023; Liang et al., 2024), and electroacupuncture (EA) reduces Alzheimer’s pathology and the worsening of this condition due to HFD (Xu et al., 2022; Zhang et al., 2024). Acupuncture has demonstrated efficacy in lowering blood glucose levels, enhancing insulin resistance, reducing the need for hypoglycemic medications or insulin, alleviating drug side effects, preventing pancreatic β-cell apoptosis, and regulating insulin signaling pathways (Xu et al., 2022; Zhang et al., 2024). Acupuncture has been proven through meta-analyses to effectively decrease fasting blood glucose (FBG) and enhance IR, making it a viable complementary therapy to hypoglycemic agents (Li S. Q. et al., 2022).

Although electroacupuncture (EA) has been demonstrated to improve glucose metabolism and lipid profiles in T2DM, the underlying mechanisms remain incompletely understood. Increasing evidence suggests that the gut microbiota plays a pivotal role in regulating host metabolism, energy homeostasis, immune responses (Sanna et al., 2019), and neuroinflammation, and dysbiosis has been implicated in both metabolic disorders and neurodegenerative diseases. Alterations in gut microbial composition have been closely associated with obesity, insulin resistance, T2DM (Sanna et al., 2019; Que et al., 2021), and Alzheimer’s disease (de Vos et al., 2022), suggesting that the gut-brain-metabolism axis may represent a critical pathway through which EA exerts systemic effects (Duranti et al., 2017; Weingarden and Vaughn, 2017).

Previous studies have reported that HFD exposure exacerbates cognitive impairment and AD-like pathology in 3xTg-AD mice, while EA intervention can alleviate metabolic dysfunction and neurodegenerative changes. However, most existing studies have focused either on metabolic outcomes or neurological pathology in isolation. Whether EA modulates gut microbiota differently in AD-prone versus wild-type individuals under metabolic stress remains largely unexplored. It is unclear whether AD-related pathological background alters the responsiveness of gut microbiota to EA intervention and consequently attenuates its metabolic benefits.

Therefore, a critical knowledge gap exists regarding the comparative effects of EA on gut microbiota composition and metabolic regulation in AD and non-AD contexts. Addressing this gap is essential for understanding inter-individual variability in EA efficacy and for optimizing acupuncture-based interventions in populations with neurodegenerative comorbidities.

In the present study, we employed HFD-induced T2DM models in both wild-type C57BL/6J mice and triple-transgenic AD (3xTg-AD, co-express human MAPT, APP, and PSEN-1 transgenes) mice to systematically compare the metabolic outcomes and gut microbiota alterations following EA intervention at the ST36 acupoint. By integrating metabolic phenotyping with 16S rRNA gene sequencing, this study aimed to (i) evaluate whether EA differentially improves glucose and lipid metabolism in AD versus non-AD mice, and (ii) elucidate distinct gut microbiota signatures associated with EA responsiveness. Our findings provide mechanistic insights into the gut microbiota-mediated effects of EA and highlight the influence of AD pathology on metabolic intervention efficacy.

## 2 Materials and methods

### 2.1 Mice

Female triple-transgenic AD (3xTg-AD) mice (Stock No. 033930; Jackson Laboratory), were obtained from the Shenzhen Center for Disease Control and Prevention, Wildtype C57BL/6J female mice were obtained from Guangzhou University of Chinese Medicine. They were maintained under standardized conditions, including a 12-h light/dark cycle and *ad libitum* access to food and water. At 7 months, during the early presymptomatic phase of AD pathology [23]—3xTg-AD mice were randomly allocated to 3X-ND, 3X-HFD, and 3X-EA groups. Age-matched female C57BL/6J mice served as the wild-type control (WT), randomly allocated to C57-ND, C57-HFD, and C57-EA groups. Animals were fed by normal diet (ND: 18% kcal from fat, 3.1 kcal/g; Jiangsu Xietong Pharmaceutical Bio-engineering Co., Ltd.) or a high-fat diet [HFD: 60% kcal from fat (31% lard, 3.7% soybean oil), 20% kcal from protein, 20% from carbohydrates; XTHF60, Jiangsu Xietong Pharmaceutical Bio-engineering Co., Ltd.] for 16 weeks. This HFD duration was chosen based on previous research demonstrating its effectiveness in inducing metabolic issues and cognitive impairments in 3xTg-AD models.

All procedures adhered to ARRIVE guidelines and received approval from the Animal Care and Use Committee of Guangzhou University of Chinese Medicine (Approval No. 20230526004).

### 2.2 EA treatment

Mice received EA treatment during the entire 4-month HFD regimen. Stainless-steel needles were inserted 2 mm deep and connected to an EA device (2 Hz, 1 mA, 20 min/session, 5 sessions/week). Bilateral ST36 (Zusanli) acupoints, located using established methods (WHO Regional Office for the Western Pacific, 2022; Medicine NAoTC, 2021), were prepared by cleansing with alcohol. ST36 acupoints, situated 0.3 cm below the fibular head at the muscle gap, posterior lateral to the knee joint, were needled to a depth of 2 mm. Mice were anesthetized using 2% isoflurane (RWD Life Science, R510-22) and positioned in a stereotaxic apparatus (RWD Life Science, 71,000). An electrical stimulator (Huatuo SDZ, China) was attached to the needle handles, administering the parameters for a duration of 16 weeks.

### 2.3 Body weight

Before the intervention and at the 8th and 16th weeks of the experiment, the body weight of mice in all groups was assessed.

### 2.4 Glucose tolerance test

GTT was assessed during the 8th and 16th weeks of the experiment. Following a 12-h fast, fasting blood glucose (FBG) levels were measured using a blood glucose meter, and each mouse received a 2.0 g/kg glucose solution. Blood glucose levels were assessed at 30, 60, 90, and 120 min after administration, and the area under the curve (AUC) for the glucose response was calculated.

### 2.5 Detection of serum lipids

Mice were anesthetized using avertin and euthanized via cervical dislocation. Blood was drawn from the abdominal aorta and allowed to sit at room temperature for 2 h. It was then centrifuged at 3,000 rpm for 15 min at 4°C. Serum total cholesterol (TC), triglyceride (TG), low-density lipoprotein cholesterol (LDL-c), and high-density lipoprotein cholesterol (HDL-c) were measured by measurement kit (Jiancheng Bio, Nanjing, China).

### 2.6 16 S rRNA amplicon sequencing experimental method

Post-experiment, mouse fecal samples were collected in a sterile stool collection box and stored in a sterile EP tube at –80°C.

The library sequencing and data processing were conducted by OE Biotech Co., Ltd. (Shanghai, China). Raw sequencing data were in FASTQ format. Paired-end reads were then preprocessed using the Cutadapt software to detect and cut off the adapter. After trimming, paired-end reads were filtered for low-quality sequences, denoised, merged, and detected, and cut off the chimera reads using DADA2 with the default parameters of QllME2. At last, the software outputs the representative reads and the ASV abundance table. The representative read of each ASV was selected using the QllME2 package. All representative reads were annotated and blasted against the Silva database (Version 138) using q2-feature-classifier with the default parameters. Total genomic DNA was extracted using the MagPure Soil DNA LQ Kit (Magan) following the manufacturer’s instructions. DNA concentration and integrity were evaluated using a NanoDrop 2000 (Thermo Fisher Scientific, United States) and agarose gel electrophoresis. The extracted DNA was kept at –20°C until it was processed further. PCR amplification of bacterial 16S rRNA genes was conducted using extracted DNA as a template, employing barcoded primers and Takara Ex Taq (Takara). Bacterial diversity was analyzed by amplifying the V3-V4 regions of 16S rRNA genes using universal primers 343F and 798R.

PICRUSt2 software (2.3.0b0) was used to predict the composition of known microbial gene functions, calculate the functional differences between different groups based on the R package Kruskal Wallis statistical algorithm, and use the R package ggplot2 tool for plotting.

### 2.7 Statistical analysis

All statistical analyses were performed using GraphPad Prism version 8.0 (GraphPad Software, San Diego, CA, United States) and R software (version 4.2.0). Data are presented as mean ± standard deviation (SD). Before statistical testing, data normality was assessed using the Shapiro–Wilk test. For normally distributed data, comparisons among multiple groups were conducted using one-way analysis of variance (ANOVA), followed by least significant difference (LSD) or Bonferroni *post-hoc* tests, as appropriate. For non-normally distributed data, the Kruskal–Wallis test was applied, followed by Dunn’s multiple comparisons test. For comparisons between two independent groups, Student’s *t*-test or Mann–Whitney U test was used depending on data distribution. Glucose tolerance test (GTT) results were analyzed by calculating the area under the curve (AUC) using the trapezoidal method.

Microbiota alpha-diversity indices were compared using the Mann–Whitney U test. Beta-diversity was assessed based on Binary Jaccard distance matrices and visualized by principal coordinates analysis (PCoA). Group differences in beta-diversity were evaluated using permutational multivariate analysis of variance (PERMANOVA) with 999 permutations. Differential taxonomic abundance analyses were performed using Metastats with false discovery rate (FDR) correction, while functional differences were predicted using PICRUSt2 and compared using the Kruskal–ruskaltion,. All statistical tests were two-tailed, and a *P*-value < 0.05 was considered statistically significant.

## 3 Results

### 3.1 EA effectively controls the body weight gain in HFD C57 mice, but fails to control the weight gain in HFD 3xTg-AD mice

**FIGURE 1:**
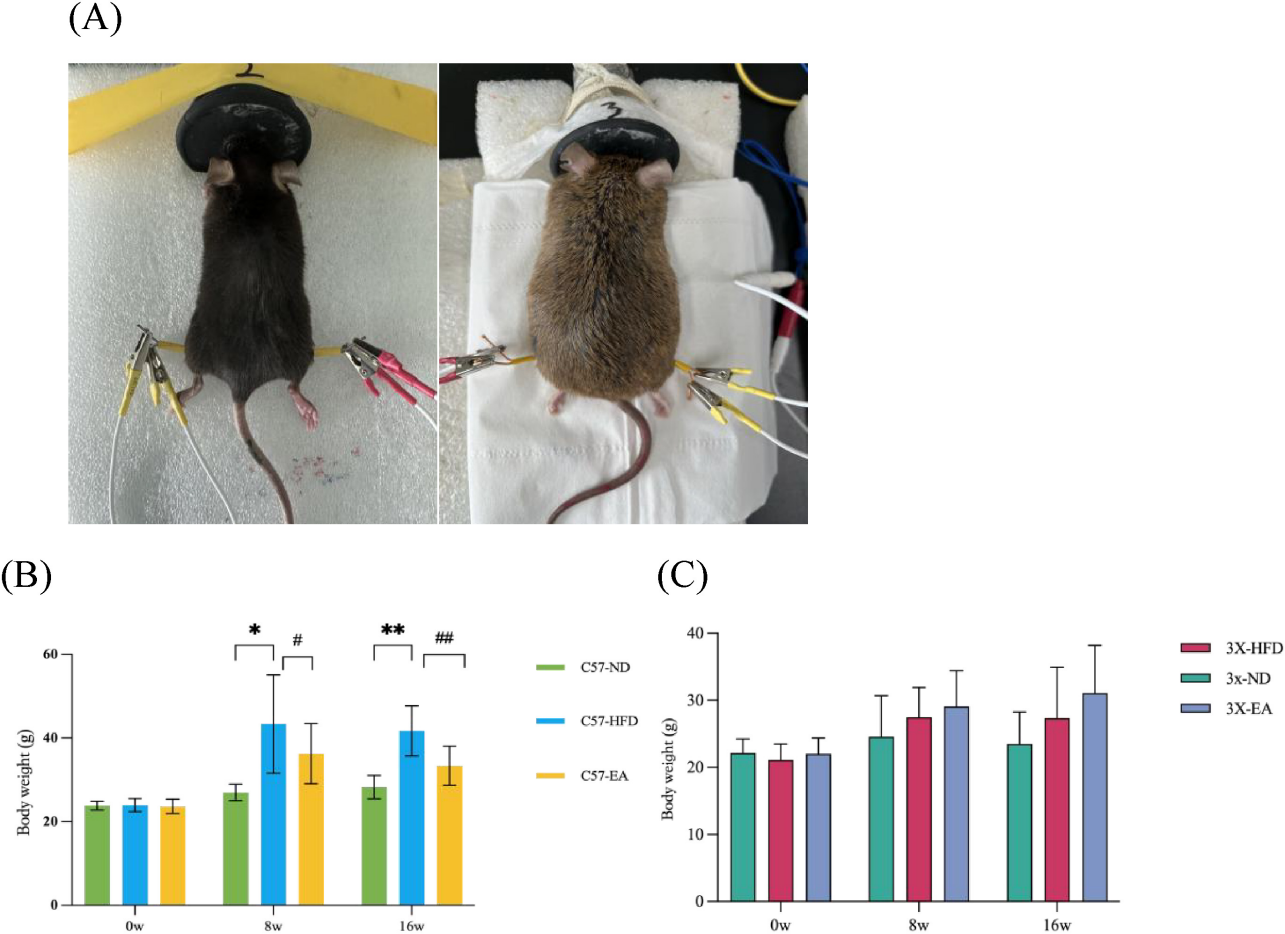
EA-ST36 alleviates HFD-induced body weight in mice. EA, electroacupuncture; ND, normal diet; HFD, high-fat diet; EA, high-fat diet and intervention of electroacupuncture. **(A)** Diagram of the EA procedure at acupoint ST36, showing needle placement and electrical stimulation setup; **(B)** effect of EA on the body weight of C57 mice; **(C)** effect of EA on the body weight of 3xTg-AD mice. *p < 0.05, **p < 0.01 versus HFD; one-way ANOVA; *n* = 6–12 per group. ^#^*P* < 0.05, ^##^*P* < 0.01.

Mice were fed either a normal or a high-fat diet for 16 weeks. Both 3xTg-AD and C57BL/6J mice on a high-fat diet exhibited significant weight gain compared to those on a normal diet (Figure 1), consistent with high-fat diet-induced obesity. The EA-ST36 treatment notably reduced weight gain in C57 mice, but this effect was not statistically significant in 3xTg-AD mice.

### 3.2 EA improves lipidemia and shows a better effect on C57 mice than 3xTg-AD mice

**FIGURE 2:**
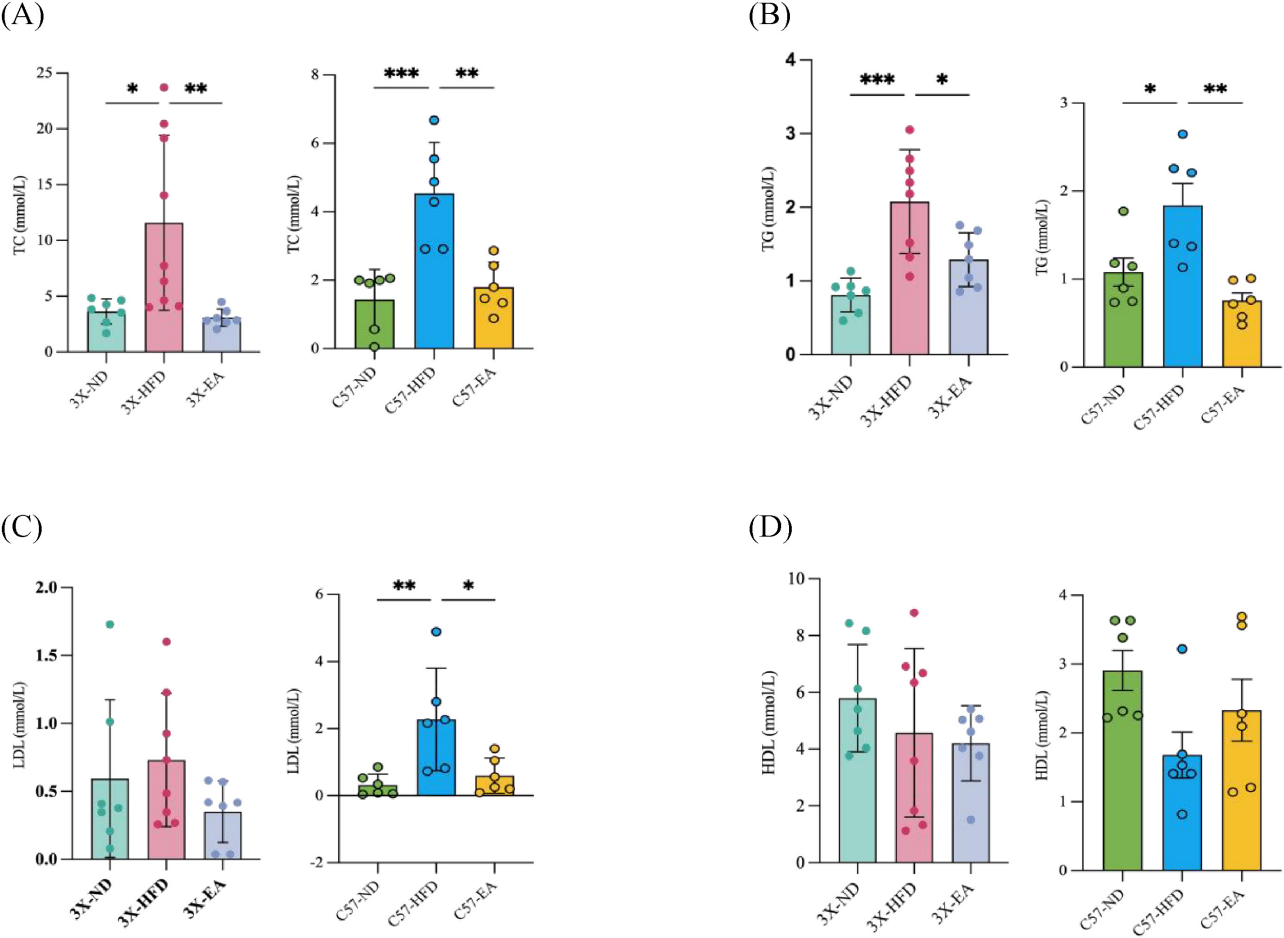
Serum biochemical parameters of mice at week 16. **(A)** Total cholesterol (TC) levels; **(B)** triglyceride (TG) levels; **(C)** low-density lipoprotein-cholesterol (LDL-C) levels; **(D)** high-density lipoprotein-cholesterol (HDL-C) levels. *p < 0.05, **p < 0.01, ***P < 0.001 versus HFD; one-way ANOVA; *n* = 6–8 per group.

As shown in Figure 2, in both 3xTg-AD mice and C57 mice, HFD feeding induced an increase in TC, TG, and LDL-C and a decrease in HDL-C, indicating lipidemia in animals. The EA-ST36 intervention significantly improved the lipidemia in both types of mice.

### 3.3 EA showed hypoglycemic in 16-week period, and show better effect on C57 mice than 3xTg-AD mice

**FIGURE 3:**
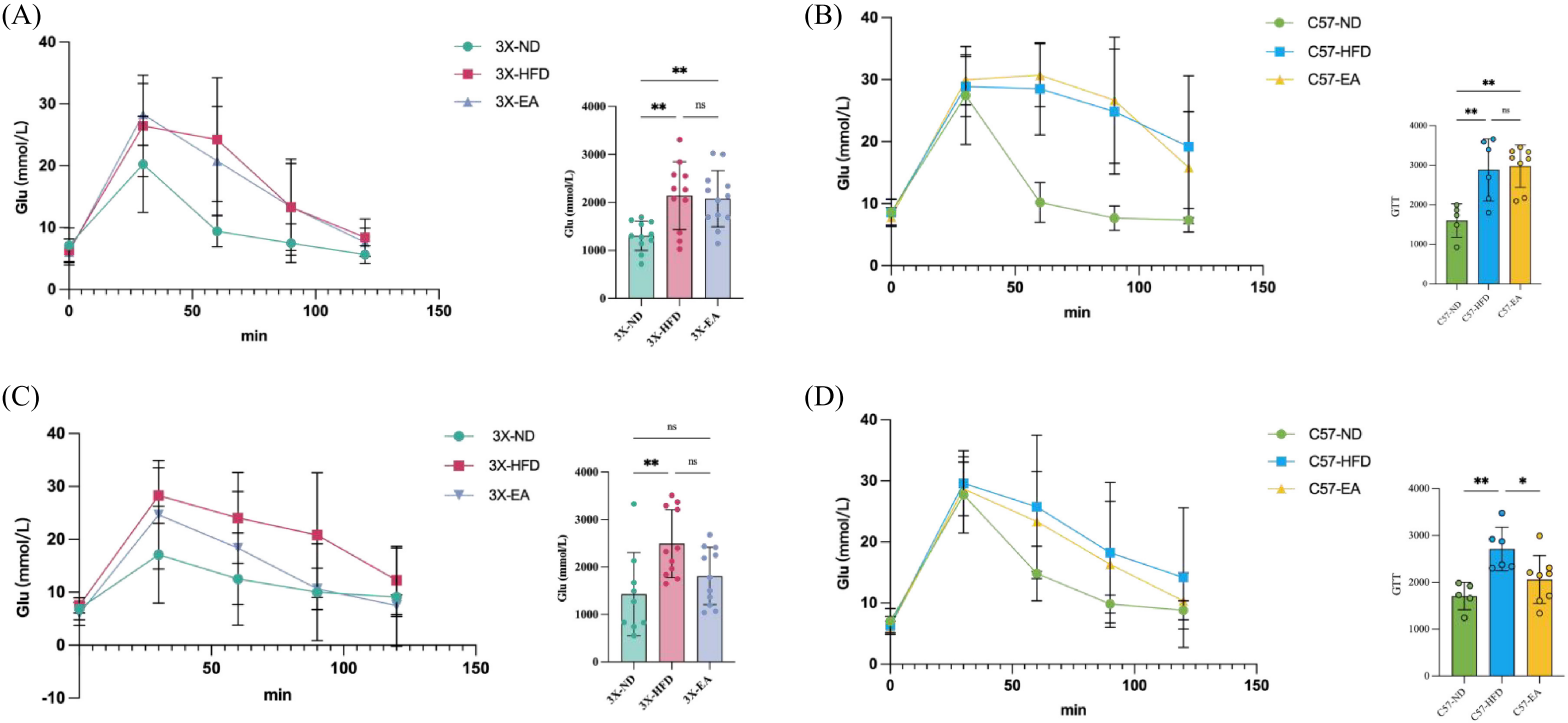
Glucose response of mice. **(A)** Glucose tolerance test (GTT) was performed on 3xTg-AD mice at the 8th week; **(B)** GTT performed on C57 mice at the 8th week; **(C)** GTT performed on 3xTg-AD mice at the 16th week; **(D)** GTT performed on C57 mice at the 16th week; *p < 0.05, **p < 0.01; one-way ANOVA. *n* = 6–12 per group.

At the 8th week of the experiment, in both 3xTg-AD mice and C57 mice, high-fat-diet fed induced impaired glucose tolerance, indicated T2DM in animals, at this timepoint, EA intervention did not show hypoglycemic effect. However, at the 16th week of the experiment, EA-ST36 intervention significantly improved the glucose tolerance, showed hypoglycemic effect, indicating a beneficial effect on metabolic dysfunction (Figure 3).

In both 3xTg-AD and C57 mice, the AUC for the HFD and EA groups was significantly higher than the ND group at the 8th week. By the 16th week, the HFD group’s AUC remained significantly higher than the ND group. However, the 3X-EA group’s AUC decreased, showing no significant difference from the 3X-ND group, while the C57-EA group’s AUC significantly decreased compared to the C57-HFD group. This suggests that EA had a beneficial effect on metabolic dysfunction over 16 weeks, with a more pronounced effect in C57 mice than in 3xTg-AD mice.

### 3.4 Impact of EA on gut microbiota

#### 3.4.1 HFD reduced abundance of ASV, and EA improved ASV counts, 3xTg-AD mice respond less effectively to EA intervention

In C57 mice, the C57-ND, C57-HFD, and C57-EA groups had unique ASV counts of 626, 198, and 234, respectively, with 266 ASV shared among the groups. In 3xTg-AD mice, the unique ASV counts for the 3X-ND, 3X-HFD, and 3X-EA groups were 551, 134, and 188, respectively.

C57-HFD group had notably lower ASV counts than the C57-ND group, EA group intervention increased the ASV counts. Moreover, there were 266 ASV in common C57-ND, C57-HFD, and C57-EA groups, and 327 ASV in common among 3X-ND, 3X-HFD, and 3X-EA groups.

**FIGURE 4:**
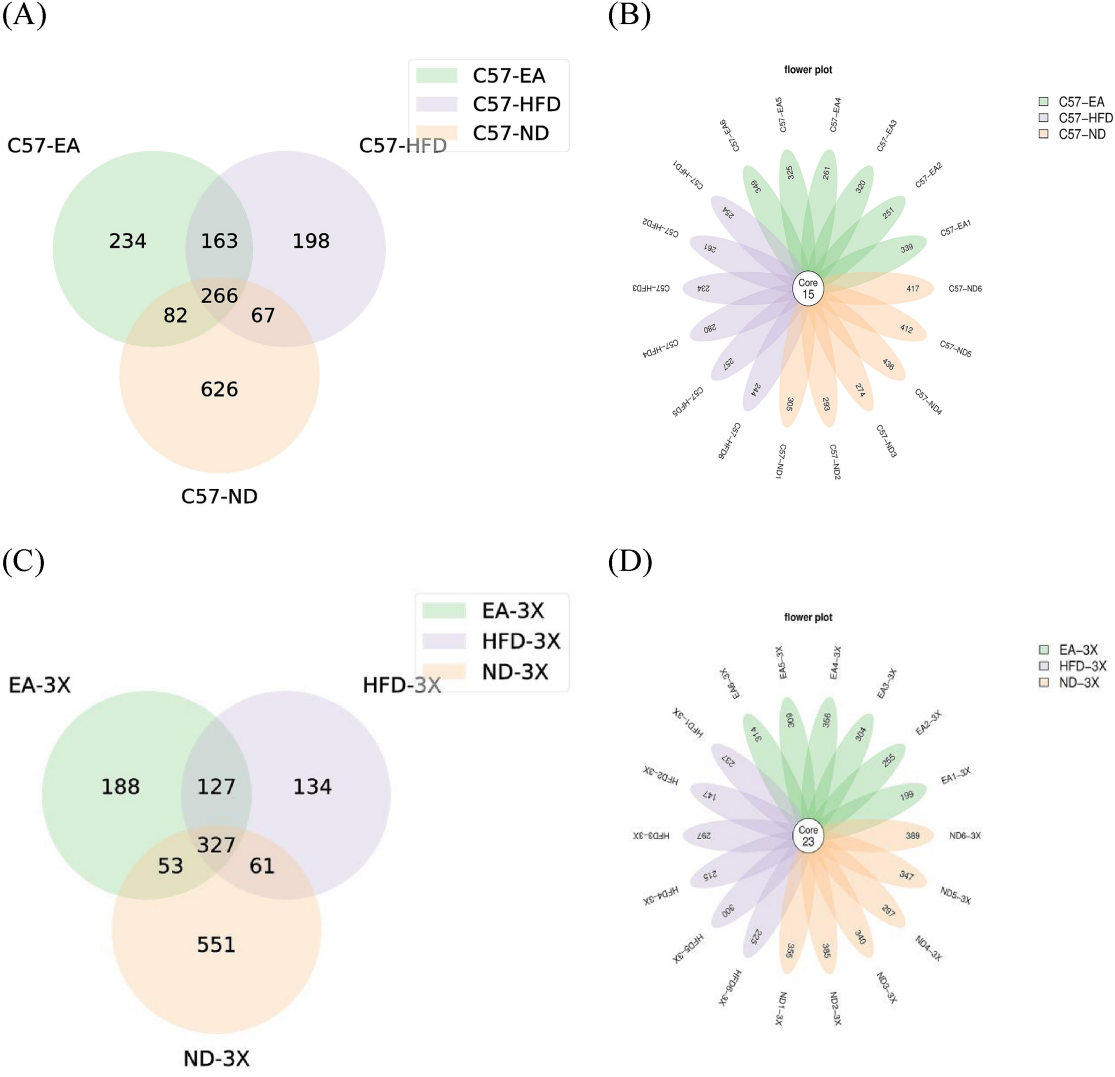
Effect of EA on the number of ASV. **(A)** Venn plot of ASV in C57 mice; **(B)** Flower plot of ASV in C57 mice;**(C)** Venn plot of ASV in 3xTg-AD mice; **(D)** flower plot of ASV in 3xTg-AD mice; *n* = 6 mice per group for microbiome analyses.

The study reveals that 3xTg-AD mice exhibit a reduced intestinal microbiota compared to C57 mice. Additionally, a high-fat diet (HFD) decreases amplicon sequence variant (ASV) counts, while EA treatment enhances ASV diversity in both mouse types. Although there were less ASV counts in 3xTg-AD mice, the common ASV among 3X-ND, 3X-HFD, and 3X-EA groups were more than the common ASV among C57-ND, C57-HFD, and C57-EA groups, indicated 3xTg-AD mice respond less effectively to EA intervention (Figure 4).

#### 3.4.2 EA therapeutic efficacy in improving α-diversity and β-diversity was impaired in 3xTg-AD mice

**FIGURE 5:**
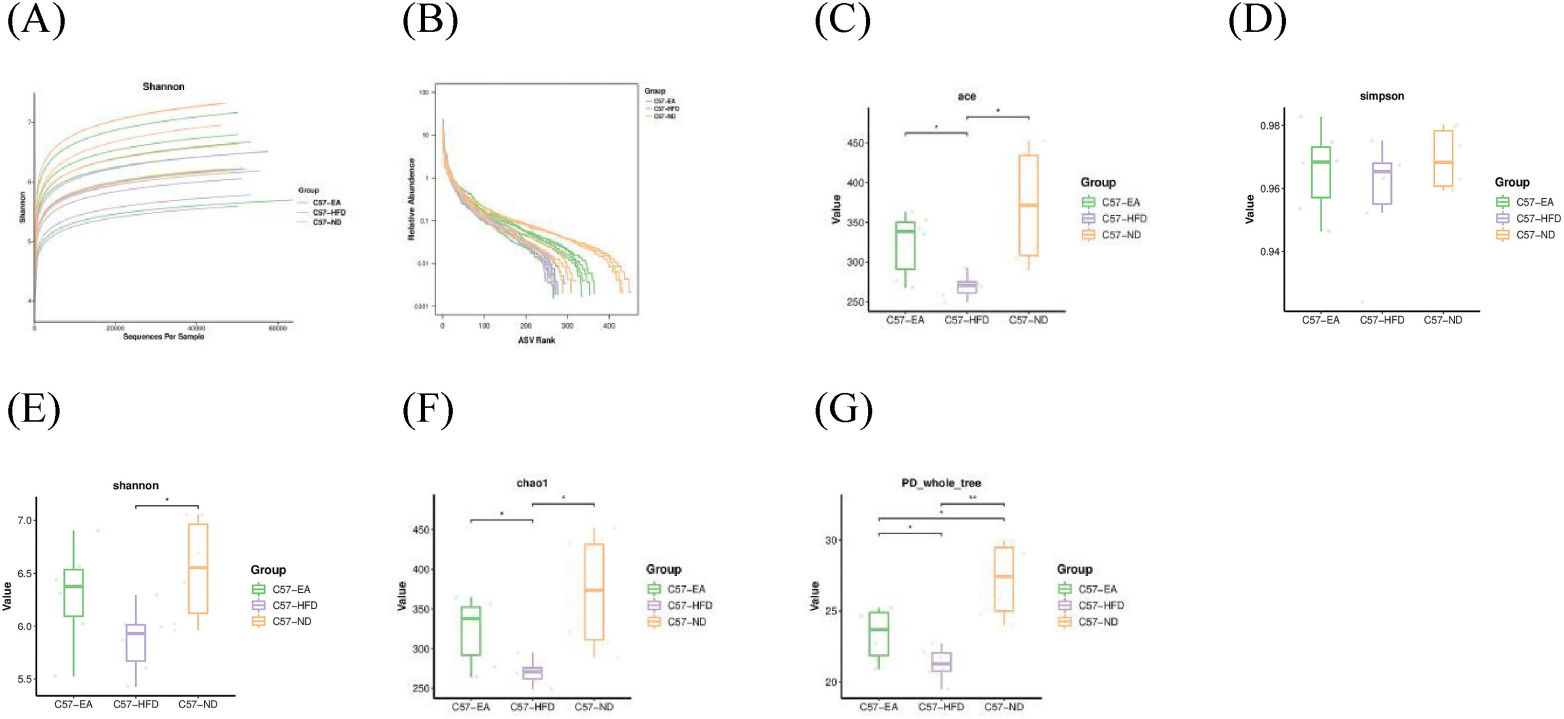
Effect of EA on the α-diversity in C57 mice **(A)** Rarefaction curve; **(B)** rank abundance; **(C)** ACE, **(D)** Simpson; **(E)** Shannon; **(F)** Chao1; **(G)** PD_whole_tree, unpaired *t*-test. *P < 0.05, **P < 0.01; *n* = 6 mice per group for microbiome analyses.

**FIGURE 6:**
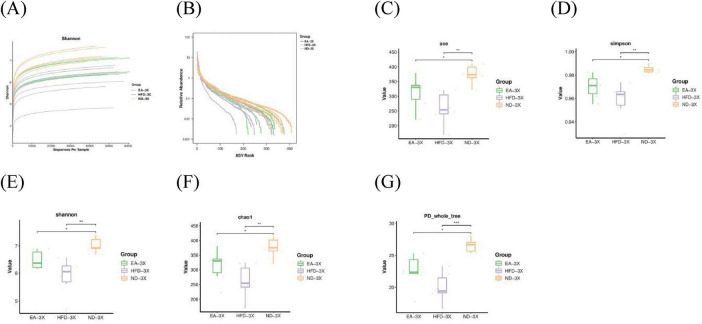
Effect of EA on theα-diversity in 3xTg-AD mice **(A)** Rarefaction curve; **(B)** rank abundance; **(C)** ACE; **(D)** Simpson; **(E)** Shannon; **(F)** Chao1; **(G)** PD_whole_tree. Unpaired *t*-test. *P < 0.05, **P < 0.01, ***P < 0.001; *n* = 6 mice per group for microbiome analyses.

Rarefaction and rank abundance curves were employed to assess sequencing depth, sample abundance, and uniformity. Figures 5A,B, 6A,B illustrate that the plateau in both curves indicates sufficient sequencing depth and consistent sample abundance.

The α-diversity index, indicating the richness and evenness of gut microbiota, is typically inversely associated with disease occurrence. Figure 5 illustrates that in C57BL/6J mice, the α-diversity indexes were significantly lower in the C57-HFD group compared to the C57-ND group (ace, C57-ND vs. C57-HFD, Mann-Whitney, U = –0.944, *P* = 0.004, FDR = 0.012). Post-EA intervention, α-diversity indexes showed significant improvement relative to the C57-HFD group (ace, C57-EA vs. C57-HFD, Mann-Whitney, U = 0.722, *P* = 0.041, FDR = 0.061), with no notable difference from the C57-ND group (ace, C57-ND vs. C57-EA, Mann-Whitney, U = –0.333, *P* = 0.393, FDR = 0.393). In 3xTg-AD mice, the α-diversity indexes were significantly lower in the 3X-HFD group compared to the 3X-ND group (ace, 3X-ND vs. 3X-HFD, Mann-Whitney, U = –0.944, *P* = 0.004, FDR = 0.012). After EA intervention, the α-diversity indexes were improved compared to the HFD group, but not significantly different (ace, 3X-EA vs. 3X-HFD, Mann-Whitney, U = 0.611, *P* = 0.093, FDR = 0.093; 3X-EA vs. 3X-ND, Mann-Whitney, U = –0.611, *P* = 0.093, FDR = 0.093) (Figure 6 and Supplementary Tables 1, 2).

These findings suggest that a high-fat diet reduces the abundance and diversity of intestinal microorganisms in mice. EA treatment enhanced α-diversity in HFD-induced T2DM mice; however, its effectiveness was diminished in 3xTg-AD mice.

**FIGURE 7:**
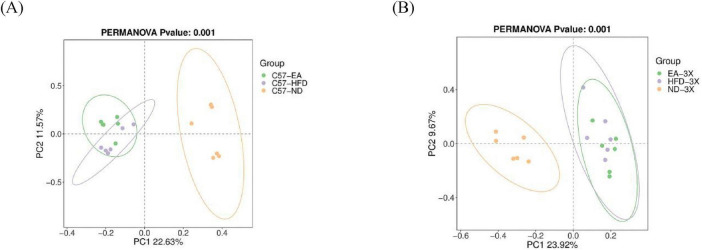
Beta-diversity was visualized by PCoA analysis. **(A)** Beta diversity of C57 mice; **(B)** beta diversity of 3xTg-AD mice; *n* = 6 mice per group for microbiome analyses.

In C57 mice, PCoA analysis using Binary Jaccard distance revealed that principal component 1 distinctly separated the C57-ND group from the C57-HFD and C57-EA groups, explaining 22.63% of the variance. However, the C57-HFD and C57-EA groups were not fully separated, with an axis contribution rate of 11.57% (Figure 7A). In 3xTg-AD mice, PCoA analysis using the Binary Jaccard distance revealed that principal component 1, accounting for 23.92% of the variance, distinguished the 3X-ND group from the 3X-HFD and 3X-EA groups. However, the 3X-HFD and 3X-EA groups were not fully separated, with an axis contribution rate of 9.6% (Figure 7B).

In C57 mice, the area where the C57-HFD and C57-EA groups partially gather overlapped (Figure 7A), the PERMANOVA analysis showed there was also significant difference between the C57-ND vs. C57-HFD group (*P* < 0.05), C57-ND vs. C57-EA group (*P* < 0.05), C57-HFD vs. C57-EA group (*P* < 0.05) in β-diversity (Table 1). In 3xTg-AD mice, the 3X-HFD and 3X-EA groups largely overlapped in the area (Figure 7B). PERMANOVA analysis indicated a significant difference in β-diversity between the 3X-ND group and the other two groups (*P* < 0.05), but no significant difference between the 3X-HFD and 3X-EA groups (Table 1).

**TABLE 1:** PERMANOVA analysis of gut microbiota based on Binary jaccard distance (*n* = 6).

Groups compared	*P*-value
C57-ND vs. C57-HFD(185)	0.005**
C57-ND vs. C57-EA(186)	0.003**
C57-HFD vs. C57-EA(187)	0.019*
3X-ND vs. 3X-HFD (189)	0.001***
3X-ND vs. 3X-EA(190)	0.002**
3X-HFD vs. 3X-EA(191)	0.458

*P < 0.05,

**P < 0.01,

***P < 0.001.

The study reveals that a high-fat diet significantly alters the microbiome profile compared to a normal diet (*P* < 0.05). Additionally, EA treatment leads to notable differences in the microbiome between C57-EA and C57-HFD groups (*P* < 0.05), whereas the β-diversity analysis indicates no significant microbiota change between 3X-EA and 3X-HFD, suggesting EA’s effect is minimal in AD mice (Figure 7).

#### 3.4.3 Changes in fecal microbiota composition following EA treatment

**FIGURE 8:**
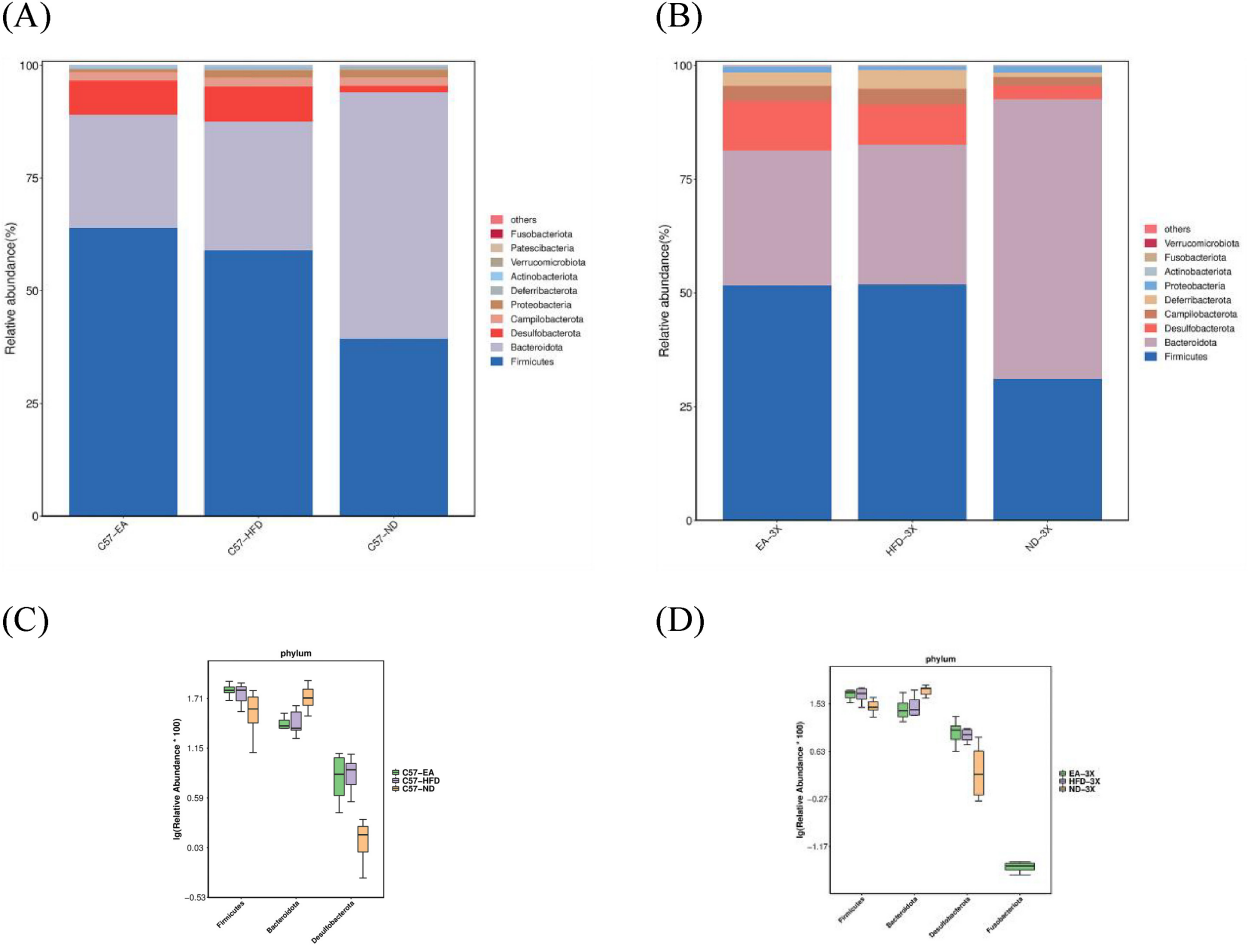
Alterations in gut microbiota after EA intervention at the phylum level. **(A)** Bar plot of gut microbiota composition in C57 mice; **(B)** bar plot of gut microbiota composition in 3xTg-AD mice; **(C)** in C57 mice, the relative abundance of *Bacteroidota* was increased and *Desulfobacterota* was decreased after EA by Metastas analysis; **(D)** in 3xTg-AD mice, EA didn’t inverse change by HFD by Metastas analysis; *n* = 6 mice per group for microbiome analyses.

**FIGURE 9:**
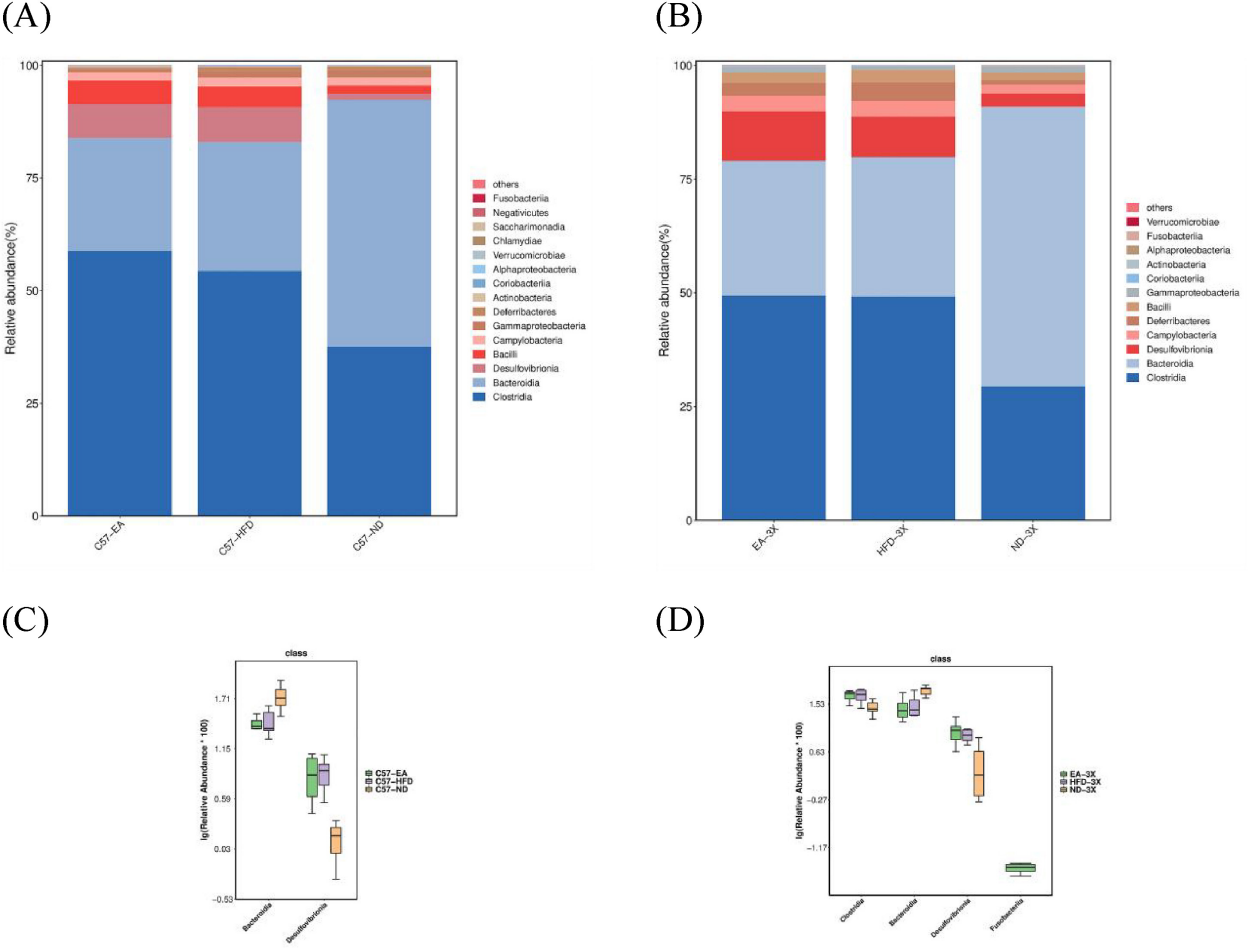
Alterations in gut microbiota after EA intervention at the class level. **(A)** Bar plot of gut microbiota composition in C57 mice; **(B)** bar plot of gut microbiota composition in 3xTg-AD mice; **(C)** in C57 mice, the relative abundance of *Bacteroidota* was increased and *Desulfobacterota* was decreased after EA by Metastas analysis; **(D)** in 3xTg-AD mice, EA didn’t reverse the change caused by HFD by Metastas analysis; *n* = 6 mice per group for microbiome analyses.

**FIGURE 10:**
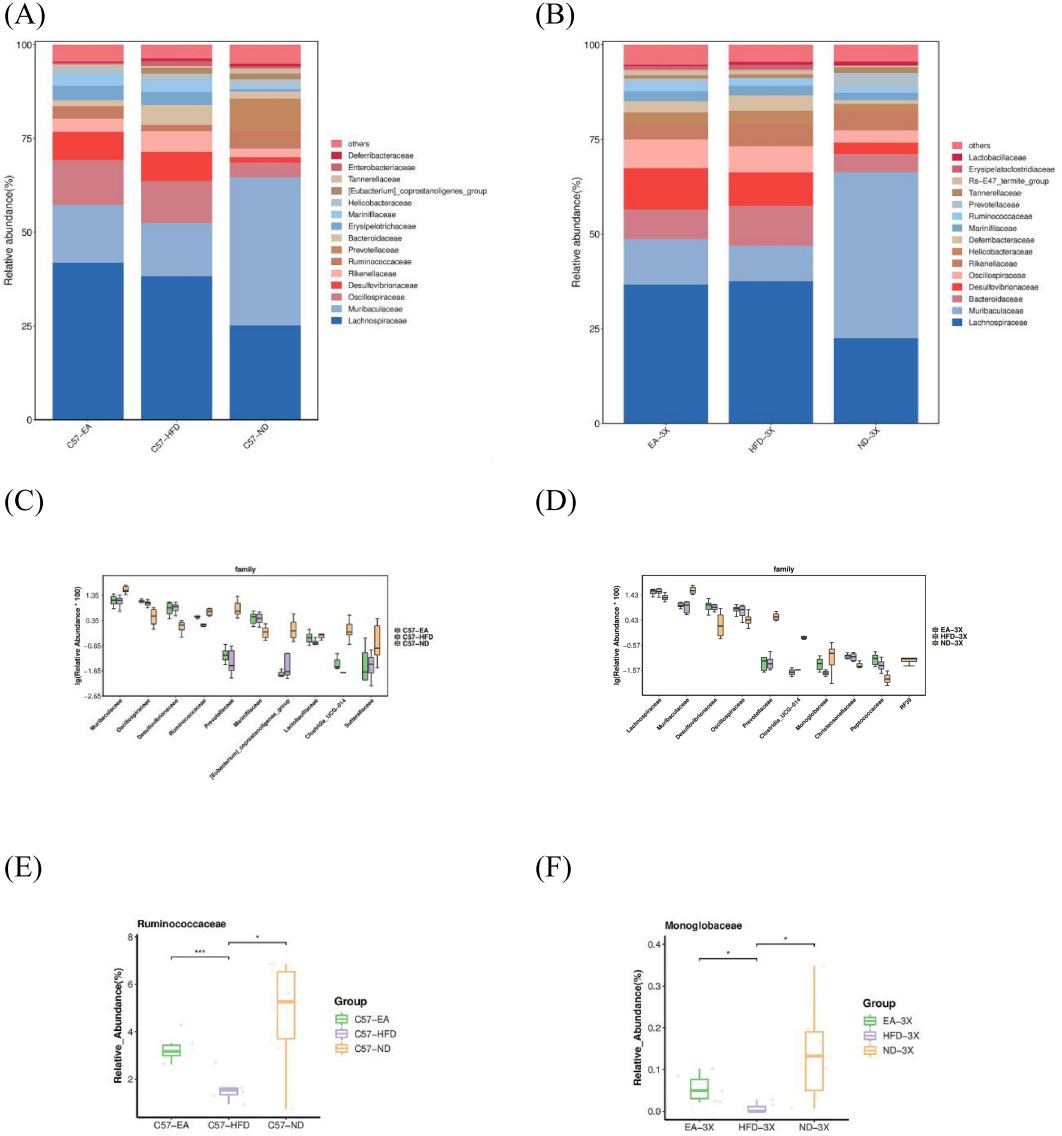
Alterations in gut microbiota after EA intervention at the family level. **(A)** Bar plot of gut microbiota composition in C57 mice; **(B)** bar plot of gut microbiota composition in 3xTg-AD mice; **(C)** in C57 mice, the relative abundance of *Ruminococcus, Prevotellaceae, Lactobacillus, Clostriodia_Ucg-014* was increased after EA by Metastas analysis; **(D)** in 3xTg-AD mice, the relative abundance of *Prevotellaceae an*d Monoglobaceae increased after EA by Metastas analysis; **(E)** EA increased *Ruminococcaceae* significantly in C57; **(F)** EA increased *Monoglobaceae* significantly in 3xTg-AD mice; *n* = 6 mice per group for microbiome analyses. *P < 0.05, ***P < 0.001.

Figures 8–10 depict the compositional variations in gut microbiota across phylum, family, class, and genus levels.

At the phylum level, both C57 and 3xTg-AD mice predominantly harbored *Firmicutes, Bacteroidetes, Desulfobacterota*, and *Campylobacter.* However, 3xTg-AD mice exhibited a higher relative abundance of *Deferribacterota* and a lower presence of *Proteobacteria* compared to the C57 group (Figures 8A,B).

At phylum level, HFD cause *Firmicutes* and *Desulfobacterota* increase, and *Bacteroidota* to decrease, however, EA treatment decreases *Desulfobacterota* and increase *Bacteroidota* in C57-HFD mice, but not in 3xTg-AD mice (Figures 8C,D).

At the class level, Clostridia, Bacteroidetes, and Desulfovibrio were dominant in both C57 and 3xTg-AD mice. However, Campylobacter and Deferribacterota were more abundant, and Bacilli were less abundant in 3xTg-AD mice compared to C57 mice (Figures 9A,B).

At the class level, HFD causes *Desulfobacterota* to increase (C57-ND vs. C57-HFD, Mann-Whitney, *U* = 1.000, *P* = 0.002, FDR = 0.006), and *Bacteroidetes* decrease in C57 mice (C57-ND vs. C57-HFD, Mann-Whitney, *U* = –0.833, *P* = 0.015, FDR = 0.027), and EA treatment also reverses this trend. However, in 3xTg-AD mice, HFD causes *Clostridia, Desulfovibrio to* increase*, and Bacteroidetes to* decrease (3X-ND vs. 3X-HFD, Mann-Whitney, *U* = –0.777, *P* = 0.025, FDR = 0.038), but EA treatment failed to reverse this trend (Figures 9C,D).

In C57 mice, the dominant bacterial families were *Lachnospiraceae, Muribaculaceae, Oscillospira, Desulfovibrio*, and *Rikenellaceae.* In contrast, 3xTg-AD mice showed dominance of *Lachnospiraceae, Muribaculaceae, Bacteroidetes, Desulfovibrio, and Oscillospira*, indicating an increased relative abundance of *Bacteroidetes* and a decrease in *Oscillospira* and *Rikenellaceae* compared to the C57 group (Figures 10A,B).

At the family level, HFD causes *Ruminococcus, Prevotellaceae, Lactobacillus*, and *Clostriodia_Ucg-014* to decrease in C57 mice, and increases them by EA. In 3xTg-AD mice, HFD causes *Lachnospiraceae, Desulfovibrio, Oscillospira*, and *Peptococcus* to increase, *Prevotellaceae* and *Monoglobaceae* to decrease, and EA increased *Prevotellaceae* and *Monoglobaceae* (Figures 10C,D).

**FIGURE 11:**
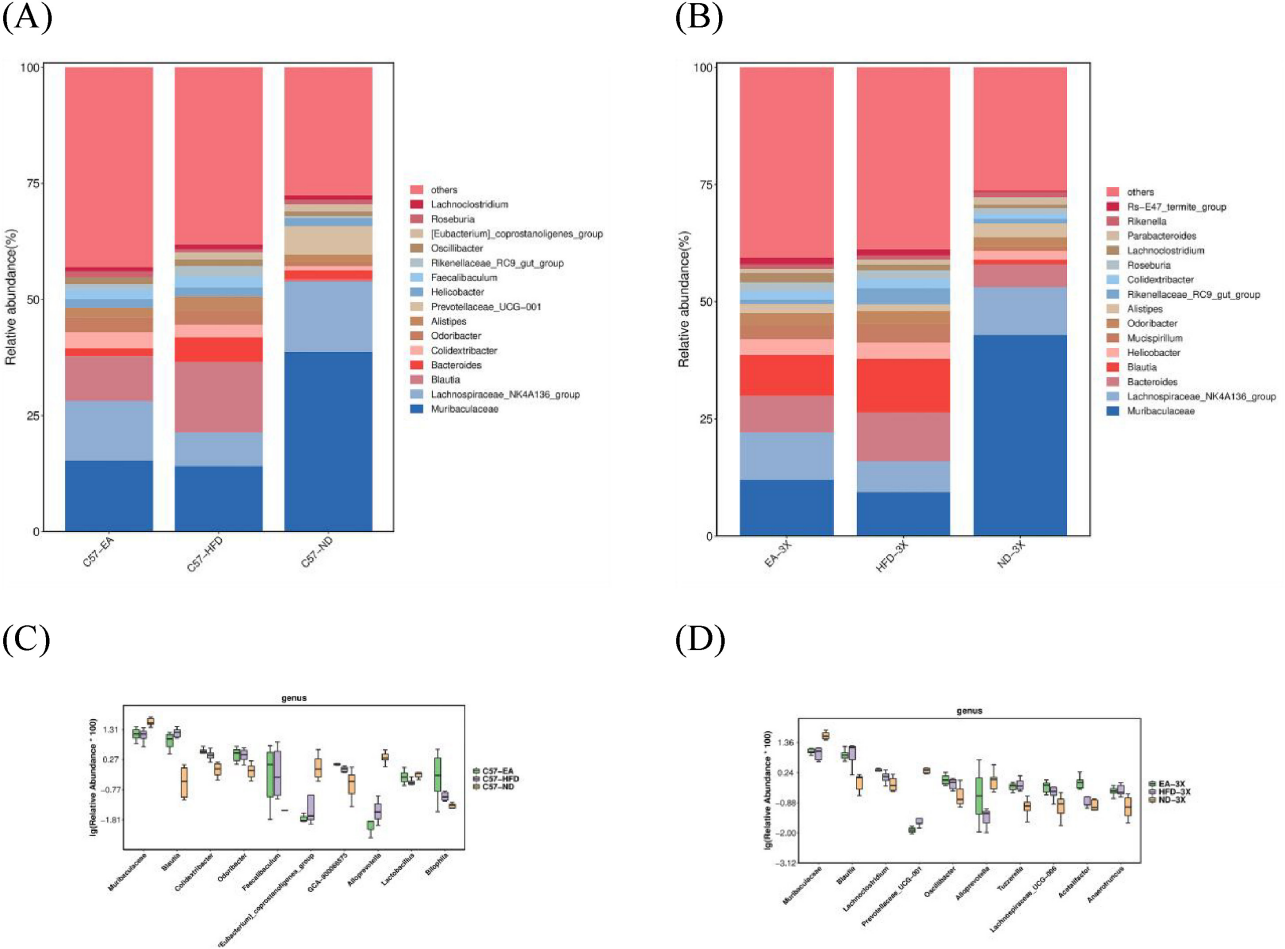
Alterations in gut microbiota after EA intervention at the genus level. **(A)** Bar plot of gut microbiota composition in C57 mice; **(B)** bar plot of gut microbiota composition in 3xTg-AD mice; **(C)** In C57 mice, the relative abundance of *Blautia* decrease and *Lactobacillus* increased after EA by Metastas analysis; **(D)** in 3xTg-AD mice, the relative abundance of *Blautia* decreased and *Alloprevotella* increased after EA by Metastas analysis; *n* = 6 mice per group for microbiome analyses.

At genus level, the five main bacterial genera in C57 mice were *Muribaculaceae, Lachnospiraceae-NK4A136, Blautia, Bacteroidetes, Colidextribacter* were dominant, while *Muribaculaceae, Lachnospiraceae-NK4A136, Bacteroidetes, Blautia, Colidextribacter* were dominant in 3xTg-AD mice. The 3xTg-AD mice showed a decrease in *Blautia* and an increase in Bacteroides relative to the C57 group (Figures 11A,B).

In C57 mice, EA elevated *Lactobacillus* levels (C57-EA vs. C57-HFD, Mann-Whitney, *U* = 0.666, *P* = 0.064, FDR = 0.092), and reduced *Blautia* abundance (C57-EA vs. C57-HFD, Mann-Whitney, *U* = –0.555, *P* = 0.132, FDR = 0.180). In 3xTg-AD mice, EA altered gut microbiota by increasing *Alloprevotella* and decreasing *Blautia* (Figures 11C,D).

#### 3.4.4 KEGG enrichment analysis

**FIGURE 12:**
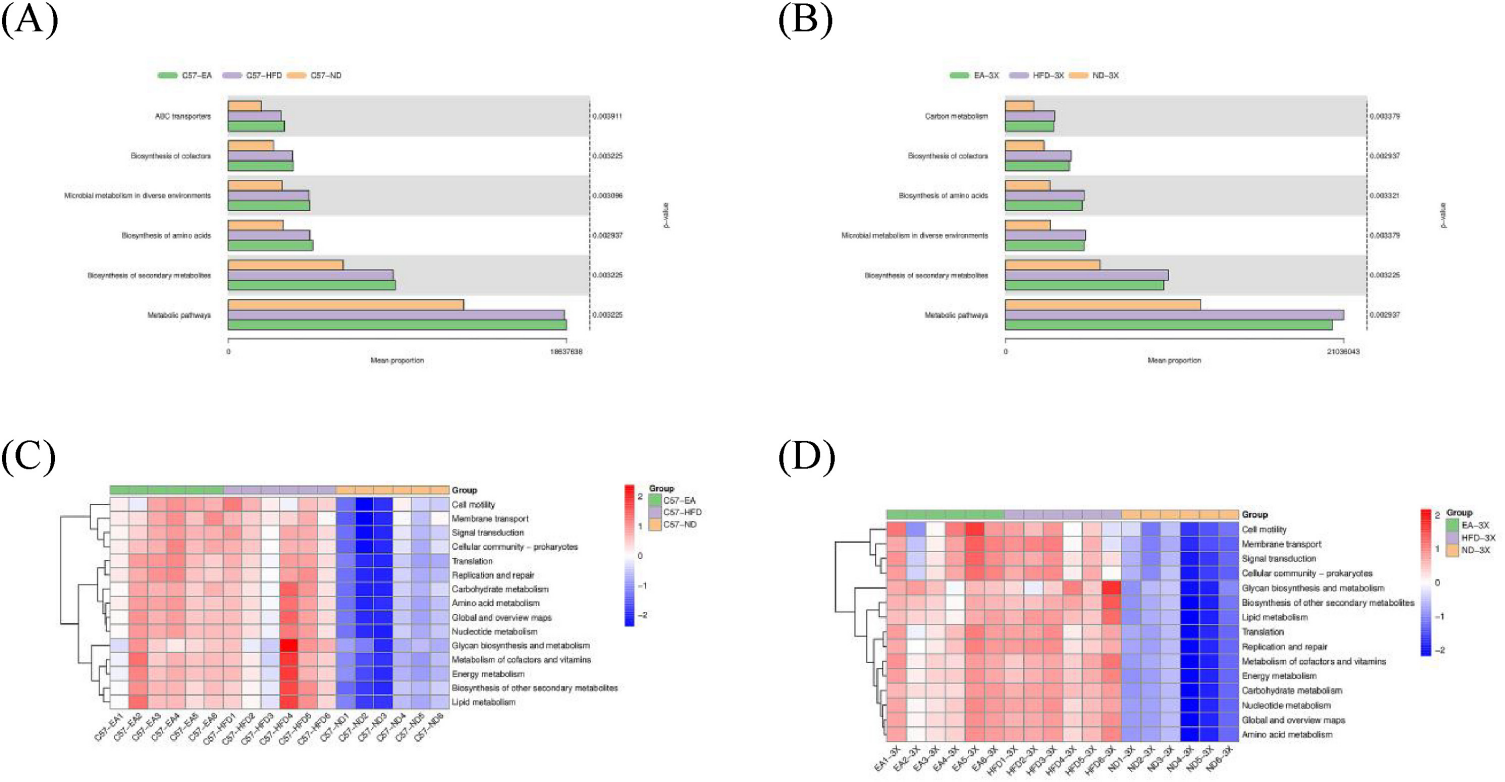
Kyoto encyclopedia of genes and genomes (KEGG). Bar chart **(A)** and heatmap **(C)** of KEGG pathways in C57 mice; bar chart **(B)** and heatmap **(D)** of KEGG pathways in 3xTg-AD mice. *N* = 6 mice per group for microbiome analyses.

To gain deeper insights into the biological processes triggered by HFD and EA, we conducted a KEGG enrichment analysis to identify the signaling pathways. KEGG enrichment analysis showed HFD induced alterations in genes involved in metabolic pathways (*P* = 0.000, FDR = 0.004), biosynthesis of secondary metabolites (*P* = 0.000, FDR = 0.004), biosynthesis of amino acids (*P* = 0.001, FDR = 0.005), microbial metabolism in diverse environment (*P* = 0.000, FDR = 0.004), biosynthesis of cofactor (*P* = 0.000, FDR = 0.004), ABC transporter (*P* = 0.019, FDR = 0.036), Two-component system (*P* = 0.003,FDR = 0.010) and Purine metabolism (*P* = 0.00, FDR = 0.004), Carbon metabolism (*P* = 0.000,FDR = 0.004), Ribosome (*P* = 0.000, FDR = 0.004) in both C57 and 3Xtg-AD mice (Figure 12). However, EA intervention induced alterations in genes involved in non-homologous end-joining pathway in C57 group (*P* = 0.017, FDR = 0.996), and primary immunodeficiency in 3xTg-AD mice (*P* = 0.04, FDR = 0.974).

## 4 Discussion

### 4.1 EA has the potential to enhance lipid profiles and lower blood glucose levels in T2DM mice

The study demonstrated that EA treatment in HFD-induced T2DM C57 mice significantly decreased body weight, serum TC, TG, LDL-C levels, and blood glucose levels, while enhancing HDL-C levels. While in HFD-induced T2DM 3xTg-AD mice, EA treatment also significantly reduced serum TC, TG levels, but LDL-C decreased and HDL-C improved not significantly, and EA failed to control body weight growth and blood glucose levels.

The metabolic benefits of EA observed in this study are consistent with previous reports demonstrating its hypoglycemic and lipid-regulatory effects in animal models and clinical populations with T2DM. Prior studies have shown that EA stimulation at ST36 improves fasting blood glucose, insulin sensitivity, and lipid metabolism through neural and hormonal pathways (Cao et al., 2017; Zhang et al., 2021; Xu et al., 2022; Zhuang et al., 2022). Our findings extend these observations by demonstrating that long-term EA intervention significantly improves glucose tolerance and dyslipidemia in HFD-induced T2DM mice; however, the above effect of EA was impaired in the 3xTg-AD mice. Notably, while earlier studies primarily focused on metabolically healthy or non-neurodegenerative models, the present study directly compares EA efficacy between wild-type and Alzheimer’s disease-prone mice. This comparative design reveals that AD-related pathological background attenuates the metabolic responsiveness to EA, highlighting a previously underexplored dimension of acupuncture efficacy.

### 4.2 Comparison of microbial diversity and richness in 3xTg-AD and C57 mice

Crucial to many bodily processes, the intestinal microbiota enhances immune function, aids in digestion, regulates intestinal hormones, modulates metabolism, and eliminates toxins (Sanna et al., 2019). Numerous studies emphasize a significant link between gut microbiota and the development and progression of type 2 diabetes (T2DM) (Sanna et al., 2019; Liang et al., 2020). A high-fat diet significantly reduces microbial diversity and richness, decreases fecal microbiota abundance, disrupts gut microbiota structure, and leads to microbial depletion (Josefsdottir et al., 2017; Liang et al., 2020; Jin et al., 2022).

Consistent with previous studies reporting reduced gut microbial diversity in T2DM and HFD-fed models (Wang et al., 2020; Gong et al., 2021; An et al., 2022; Pan et al., 2023; Yue et al., 2024), we observed significant decreases in alpha-diversity indices following HFD exposure. Similar improvements in microbial richness have been reported after EA intervention in diabetic rodent models; however, our data further demonstrate that this restorative effect is markedly blunted in 3xTg-AD mice, suggesting that neurodegenerative pathology may constrain microbiota plasticity in response to EA.

### 4.3 Comparative analysis of 3xTg-AD and C57 mice composition

In 3xTg-AD mice, there was an increase in *Deferribacterota* (phylum) and *Campylobacter* (class), while *Proteobacteria* (phylum), *Bacilli* (class), *Oscillospira* and *Rikenellaceae* (family), and *Blautia* (genus) showed a decrease compared to the C57 group.

*Deferribacterota*, as pathogenic bacteria (Coffman et al., 2024), was positively correlated with increased psychological stress in mice treated with HFD (Zhang et al., 2022). *Campylobacter* is among the four main causes of gastroenteritis worldwide (Costa and Iraola, 2019) and can spread colorectal cancer (Du Toit, 2025). *Oscillospiraceae* and *Rikenellaceae* have been linked to better metabolic syndrome outcomes, decreased inflammation, and possibly beneficial effects on liver health (de Vos et al., 2022). These findings help explain why 3xTg-AD mice are susceptible to psychological disease and digestive disease.

The 3xTg-AD mouse model demonstrates age-related progression of Aβplaques, NFTs, and memory impairments (Chen et al., 2013), alongside brain hypometabolism characteristic of the pre-dementia stage and reduced bacterial diversity (Sanguinetti et al., 2018). Differences in gut microbiota composition between 3xTg-AD mice and WT mice emerged at 6 months, coinciding with disease onset. This suggests that alterations in brain state can influence gut microbiota composition, and the pathological condition of AD may contribute to these changes (Hang et al., 2022). Studying microbiome composition over time is essential to identify changes in microbial communities that impact the host (Chen et al., 2022). In AD mice, gut microbiota diversity revealed an increase in *Desulfovibrio* at the family level (Zhang et al., 2017), accompanied by decreased short-chain fatty acid levels. These microbiota alterations impact metabolic pathways, contributing to cognitive impairment, Aβdeposition, and intestinal abnormalities. In addition, the clearance of gut microbiota in AD mice is related to central Aβ levels (Harach et al., 2017). After microbiota transplantation in AD mice, an increase in Aβ accumulation was found in the brain. Study found the dissemination of AD-related pathogenic proteins through the vagus nerve led to cognitive decline (Chen et al., 2021). Vagus nerve stimulation has been approved as a new strategy for AD intervention through the brain-gut-microbiota axis (Yan et al., 2024), transcutaneous vagus nerve stimulation was reported to modulate depression-like phenotype induced by high-fat diet via P2 × 7R/NLRP3/IL-1β in the prefrontal cortex (Li et al., 2024), acupuncture at ST36 has clinical benefits in relieving inflammation through several mechanisms and pathways such as vagus nerve activation, toll-like receptor 4 (TLR4)/NF-κB signaling, macrophage polarization, mitogen-activated protein kinase (MAPK) signaling pathway, and cholinergic anti-inflammatory pathway (Oh and Kim, 2021), these findings help understanding acupuncture’s role in the present study.

### 4.4 HFD changes composition differently between 3xTg-AD mice and C57 mice

In C57 mice, HFD cause *Firmicutes, Desulfobacterota, Blautia* increase, and *Bacteroidota, Ruminococcus*, *Prevotellaceae, Lactobacillus, Clostriodia_Ucg-014* decrease. These findings are consistent with earlier research indicating that HFD-T2DM rats exhibit an increased proportion of *Blautia, Lachnospiraceae*, and *Proteobacteria*, alongside a decreased proportion of *Bacteroidetes, Lactobacillaceae*, and *Lactobacillus* (Yue et al., 2024). Studies have demonstrated that *Lactobacillus* enhances glucose and lipid metabolism in mouse models of T2DM (Lee et al., 2021; Zhong et al., 2021; Liu et al., 2022). Research shows that *Bacilli* and *Clostridiales* can inhibit NF-KB activity, reduce pro-inflammatory factors, and exhibit anti-inflammatory effects (Cunningham et al., 2021). *Prevotella* is typically considered a bacterium associated with a healthy diet and playing a role as a probiotic in the human body; however, a decrease in the Prevotella genus is associated with certain diseases. The enzymatic digestion of polysaccharides in the *Prevotella* library represents a valuable resource for optimal digestion kinetics and intestinal homeostasis. The higher the diversity of *Prevotella*, the more beneficial its microbial community’s fermentation ability is for the health of the human gut (Tett et al., 2021). Clinical studies indicate that *Lactobacillus* is associated with LDL-C levels in T2DM patients, and dietary strategies to alter intestinal *Lactobacillus* populations may benefit diabetes, hyperlipidemia, and other metabolic disorders (Liu et al., 2012). These findings indicate that targeting the intestinal microbiota could be a viable strategy for preventing and treating T2DM.

In 3xTg-AD mice, HFD causes *Lachnospiraceae, Desulfovibrio, Oscillospira*, *Peptococcus, Clostridia, and Desulfovibrio to* increase, *Bacteroidetes, Prevotellaceae* and *Monoglobaceae to* decrease. Increased levels of *Desulfovibrionales* and *Oscillospira* are positively correlated with a higher risk of AD (Zeng et al., 2023). *Desulfovibrio* (species) were more abundant in individuals who later developed diabetes (Murphy et al., 2017), and were more prevalent in the gut microbiota of Chinese individuals with T2DM, as observed in 3xTg-AD mice (Qin et al., 2012).

*Lachnospiraceae* can produce short-chain fatty acids (SCFAs), playing a role in the metabolism of various carbohydrates and fat (Zhang et al., 2021). Enhancing probiotics like *Blautia* and *Lactobacillus* while reducing opportunistic pathogens can improve intestinal acidity, suppress harmful bacterial growth, and reduce mucosal inflammation.

### 4.5 EA changes composition differently between 3xTg-AD mice and C57 mice

In C57-HFD mice, EA elevated the relative abundances of *Bacteroidota* (phylum), *Ruminococcus* (family), and *Lactobacillus* (genus), while reducing *Desulfobacterota* (phylum) and *Blautia*, with a notable increase in *Ruminococcus* (family).

The *Ruminococcaceae* family is essential for converting resistant starch into SCFAs (Kim et al., 2024). SCFAs are essential for maintaining intestinal health, regulating inflammation, and protecting against illnesses. They are inversely associated with most indicators of metabolic-associated fatty liver disease (MAFLD) and positively associated with HDL-C (Ji et al., 2024). SCFAs have been linked to AD and may enhance the glutamate-glutamine shuttle to potentially counteract oxidative damage in neurons at the cellular level. Research shows that supplementing with SCFAs in the diet during early aging can modulate neuroenergetics to ease AD, offering a hopeful path for new drug development (Doifode et al., 2021; Sun et al., 2023), acupuncture stimulated pathways associated with the synthesis of SCFAs (Qi et al., 2025).

*Ruminococcaceae* and *Prevotellaceae* are linked to combating immune diseases (Peng et al., 2019) and have shown both positive and negative associations with a growing range of intestinal and extraintestinal conditions, including inflammatory bowel diseases and neurological disorders (Crost et al., 2023). Acupuncture can be a possible treatment for various diseases through its modulation of various cytokines, leading to reduced inflammation (Lee and Kim, 2022). EA ameliorates intestinal inflammation by activating α7nAChR-mediated JAK2/STAT3 signaling pathway in postoperative ileus. The expression of GABAA receptors in DMV neurons was inhibited by EA, leading to the suppression of IM-induced inflammation via the activation of the α7nAChR-mediated JAK2/STAT3 signaling pathway (Yang et al., 2021). In experimental models, acupuncture reduced the expression of inflammatory cytokines, enhanced IL-10 expression, promoted Treg cell differentiation, and influenced macrophage polarization. Acupuncture’s ability to reduce inflammation has been proven to engage the vagal-adrenal and cholinergic anti-inflammatory pathways (Gamus and Shoenfeld, 2025). Therefore, EA may stimulate the vagus nerve to improve the inflammatory pathway and related cytokines, improve the intestinal environment and composition of gut microbiota, help enhance SCFAs synthesis and lipid metabolism, achieving the effect of improving AD through the brain-gut axis.

Research indicates that high fructose corn syrup (HFCS) intake reduces *Ruminococcaceae* levels in adolescent mice (Bhat et al., 2021), while individuals with a higher healthy eating index exhibit increased levels of *Ruminococcaceae* (Ma et al., 2021). *Lactobacillales* and *Lactobacillus* are beneficial bacteria that influence glucose absorption, regulate lipid metabolism, inhibit inflammation, reduce LPS content, and have hypoglycemic and hypolipidemic effects. They are also negatively correlated with FBG (Bhat et al., 2021). Previous research demonstrated that a High-Fat High-Sugar Diet exacerbates memory impairment in 3xTg-AD mice (Lamichhane et al., 2024). However, electroacupuncture has been shown to enhance cognitive function in these mice by increasing gut microbiota diversity and richness, modifying microbiota composition, altering the abundance of specific species such as *Lactobacillus*, and regulating the functional metabolism of the gut microbiota (Xu et al., 2025). *Ruminococcaceae* was exclusively found in 3xTg-AD mice, with a high-fat diet enhancing this effect. The resulting profiles could indicate predictive or risk-stratifying potential by appearing before overt cognitive impairment (Sanguinetti et al., 2018).

In 3xTg-AD mice, EA treatment altered gut microbiota by increasing the relative abundances of *Monoglobaceae* and *Alloprevotella*, while reducing *Blautia* (Figures 11C,D), among which EA can increase the family. Monoglobaceae was found as beneficial bacteria (Chen et al., 2025), and the enrichment of *Monolobaceae* is positively correlated with the production of indole-3-propionic acid (IPA) (Hu et al., 2025). As an important microbial tryptophan metabolite from intestinal sources, IPA has been proven to have various biological activities, including anti-inflammatory, antioxidant, and immunomodulatory effects; conversely, lower relative abundances of the family *Monoglobaceae* were correlated with mental disease (Li H. et al., 2022; Kim et al., 2023). In patients with irritable bowel syndrome (IBS), the relative abundances of potentially beneficial bacteria, including *Monoglobaceae, Lachnospiraceae*, and Ruminococcaceae, were reduced (Paripati et al., 2023). *Alloprevotella*, known for producing short-chain fatty acids, has been shown to ameliorate autoimmune diseases through the microbiota-metabolites-immunity axis (Han et al., 2024). Experiments have shown that both short-term and long-term SCFA treatment can significantly improve cognitive impairment, and is accompanied by changes in the composition of the gut microbiota, as the beneficial gut microbiota *Alloprevotella* is positively correlated with hippocampal volume (Lee et al., 2025). This suggests that EA may enhance the treatment of autoimmune and psychiatric disorders by modulating *Monoglobaceae* and *Alloprevotella*. The study observed that following EA intervention, there was an increase in *Firmicutes* abundance, a decrease in *Bacteroidetes*, and a reduction in serum levels of LPS and TNF-α (Wang et al., 2022). EA treatment led to an increase in beneficial bacteria, such as *Lactobacillus* and *Firmicutes*, while reducing the proportion of opportunistic bacteria.

EA can typically affect body weight and serum lipid metabolism, along with notable alterations in the gut microbiota. These results suggest that the two animals might respond to EA differently. Previous studies have reported that EA modulates gut microbiota composition by increasing short-chain fatty acid-producing bacteria and reducing opportunistic pathogens, thereby improving metabolic and inflammatory outcomes. In agreement with these findings, our results show that EA increased the relative abundance of *Ruminococcaceae* and *Lactobacillus* in C57 mice, taxa commonly associated with improved glucose metabolism and lipid homeostasis.

Importantly, the present study reveals a differential microbial response to EA in 3xTg-AD mice, characterized by enrichment of *Monoglobaceae* and *Alloprevotella* rather than classical metabolic-beneficial taxa observed in wild-type mice. This divergence suggests that EA may engage distinct microbiota-mediated pathways depending on the host’s neurological and metabolic background, an aspect not previously emphasized in acupuncture–microbiome research.

### 4.6 Translational relevance and feasibility of electroacupuncture in humans

Electroacupuncture (EA) has been widely used in clinical practice for metabolic and neurological disorders, supporting the translational relevance of the present findings. Clinical studies have shown that EA, particularly at the ST36 acupoint, can improve glucose metabolism, insulin sensitivity, and lipid profiles in patients with type 2 diabetes mellitus, with a favorable safety profile and minimal adverse effects.

Importantly, the current findings suggest that the therapeutic efficacy of EA may vary depending on underlying neurological pathology. While EA robustly improved metabolic outcomes and gut microbiota composition in wild-type mice, its effects were attenuated in 3xTg-AD mice. This observation may have direct clinical implications, indicating that patients with Alzheimer’s disease or preclinical neurodegeneration could exhibit reduced responsiveness to EA-based metabolic interventions. Such variability highlights the importance of patient stratification and personalized treatment strategies in future clinical applications.

Furthermore, the gut microbiota-mediated effects observed in this study align with emerging clinical evidence linking EA to microbiome modulation in humans. Given the growing recognition of the gut–brain–metabolism axis in metabolic and neurodegenerative diseases, EA may serve as a promising non-pharmacological approach to simultaneously target metabolic dysfunction and neuroinflammation. However, longitudinal clinical studies integrating metabolic parameters, cognitive assessments, and gut microbiota profiling are required to validate these translational implications.

Overall, while caution is warranted when extrapolating animal findings directly to humans, the present study provides mechanistic support for the clinical use of EA in metabolic disorders and underscores the need to consider neurological comorbidities when evaluating therapeutic outcomes.

### 4.7 Limitations and implications for future studies

There are several limitations in the current study. Our findings suggest that EA affects gut microbiota and supports glucose metabolism; however, the specific contribution of individual bacteria to EA’s therapeutic effects remains unexplored. In the future, gut microbiota gene knockout, fecal microbiota transplantation techniques can identify bacteria or microbial products responsible for EA’s hypoglycemic effects. Second, AD pathology indicated by Aβ or tau expression has not been measured in this study and requires deeper investigation in pathology in the future. Third, sex bias exists in this study since only female mice were included. Fourth, the sample size (*n* = 6) was relatively small and may limit statistical power.

## 5 Conclusion

EA notably enhanced glucose metabolism and lipid profiles in T2DM C57 mice, but its therapeutic effect was diminished in 3xTg-AD mice. Variations in gut microbiota regulation in 3xTg-AD mice may account for their response to EA treatment.

## Data Availability

The original contributions presented in the study are included in the article/Supplementary material, further inquiries can be directed to the corresponding author.
